# Lived experience narratives in health professional education: educators’ perspectives of a co-designed, online mental health education resource

**DOI:** 10.1186/s12909-023-04956-0

**Published:** 2023-12-12

**Authors:** Tracey Parnell, Kate Fiske, Kellie Stastny, Sarah Sewell, Melissa Nott

**Affiliations:** 1 School of Allied Health, Exercise and Sports Sciences, Faculty of Science and Health, Charles Sturt University, PO Box 789, Albury, NSW 2640 Australia; 2 Gateway Health, 155 High Street, Wodonga, Victoria 3690 Australia; 3 Intervoice, 21 Warwick Road, Wodonga, Victoria 3690 Australia; 4 Listening to Voices, C/- 155 High Street, Wodonga, Victoria 3690 Australia; 5 Three Rivers Department of Rural Health, Charles Sturt University, Wagga Wagga, NSW 2678 Australia

**Keywords:** Lived experience, Co-production, Co-design, Mental health education, Consumer, Advocacy

## Abstract

**Introduction:**

Meaningful involvement of people with lived experience is an invaluable approach to education that facilitates the development of knowledge, skills and attitudes for collaborative, compassionate and person-centred healthcare practice. The purpose of this evaluation was to gain health professional educators’ perspectives of an online learning resource that presents the lived experiences of people who have been consumers of the Australian mental health system.

**Methods:**

A cross sectional study design was used to survey educators who had registered to use the online education resource. Data were collected using an online survey and follow-up interviews. Two lived experience researchers were involved in the research. Quantitative survey data were analysed descriptively, and qualitative data were analysed thematically.

**Findings:**

The Listening to Voices online education resource is being used in a range of settings. Educators perceived the content facilitated achievement of learning outcomes related to understanding the experiences of people with mental health issues. The free, online, and flexible design of the resource promoted access and helped overcome barriers to including lived experience experts in education. The powerful impact of the resource and importance of creating safe learning environments when using the resource were highlighted. Suggestions for future developments were provided.

**Conclusion:**

Involving people with lived experience in education of healthcare students and professionals can assist in developing skills for collaborative, compassionate, and person-centred care. Implementation of co-design principles and the use of creative pedagogical approaches can contribute to the development of impactful educational resources that foreground lived experience. Making these resources flexible and freely available online improves their utility.

**Supplementary Information:**

The online version contains supplementary material available at 10.1186/s12909-023-04956-0.

## Introduction

The involvement of people with lived and living experience in the education and ongoing professional development of health professionals, and as means of improving health outcomes is not a new phenomenon. The importance and value of involving people with lived and living experience of mental health issues has received global recognition as an approach to improving service development and delivery [1] and enhancing the education of health students and professionals [2–5]. This increased emphasis has been partly in response to the mandatory requirement of some professional bodies that people with lived experience, or consumers, be involved in the design, delivery, and evaluation of the education of health professionals (see for example: [6]). Drawing on lived experience as an educative tool has been shown to be an effective and powerful way to increase understanding and develop a deeper appreciation for the lives and experiences of other people [2, 7]. The opportunity to learn from people with lived experience supports the development of skills, knowledge, and attitudes required for effective healthcare practice [8]. Additionally, approaches to education involving people who have accessed healthcare services and systems have been identified as engaging and are positively evaluated by students [2, 9]. For the purposes of this paper, the term ‘people with lived experience’ includes people with *lived* (previous) and *living* (current) experience.

Meaningful involvement of people with lived experience in health service and curriculum design, delivery and evaluation offers benefits to learners, educators, people with lived experience and broader society [10]. Educators have a pivotal role to play in building capacity by providing health students and staff with opportunities to participate in learning activities that include engagement with people with lived experience. Specifically considering preparation to work in mental health practice, meaningful involvement of people with lived experience in the design and delivery of education can assist students to develop skills in critical thinking, communication and empathy, as well as challenge stereotypes, and address stigma and fear [2–5]. Opportunities to learn from and with people with lived experience are critical to develop future health professionals with the capability to work collaboratively and compassionately with people experiencing psychological distress or mental ill-health, their families and carers [11–13], and who can contribute to the development of a responsive, person-centred, and humane mental health system [14]. Previous research has highlighted that engaging people with lived experience provides opportunities to contextualise academic learning and better prepares students for future work experiences [14]. Whilst this learning may, at times, contradict academic content, this juxtaposition can create important opportunities for reflection, exploration, critical thinking, discussion, and debate that positively impacts students’ attitudes, and perceptions [15–17], and ultimately, their approach to healthcare delivery.

The co-creation of learning experiences which aim to capitalise on the “transformative nature of bringing together lived experience, academic and professional knowledge, skills and expertise” [18, p. 905] provides educators with meaningful opportunities for reflection on their position and privilege, and for the creation of spaces where power and influence are afforded to people with lived experience [19]. In addition, modelling collaborative engagement between people with lived experience and educators may positively influence learners’ attitudes and beliefs about people with lived experience of mental illness [11, 20].

For people with lived experience, opportunities to be actively and meaningfully involved in education have been found to contribute to self and collective efficacy, empowerment, and a sense of hope [21]. People with lived experience have reported that meaningful involvement in education can contribute to a sense of positive wellbeing [2]. In considering the benefits to people with lived experience and the broader community, Sunkel and Sartor [1, p. 161] suggested that the focus is on creation of “communities in which people with lived experience are able not only to survive but rather thrive with a mental health condition”. Positive engagement in the education of health professional students and staff may contribute to achieving this outcome.

Despite the many benefits of involving people with lived experience in the education of health professionals, there remain barriers which impact the successful and sustainable integration of this approach to education. Barriers identified in the literature include: attitudes of educators reflecting that people with lived experience are unreliable or vulnerable [22]; reluctance of educators to relinquish the gatekeeper role including moderation of content [23]; the ongoing privileging of knowledge derived from medicalised epistemology over lived experience [18]; beliefs that people with lived experience may use the opportunity to promote their own agenda which may contradict academic content [24]; limited access to training and support to assist educators to effectively engage with people with lived experience [13]; lack of access to funding for remuneration of people with lived experience involvement [25]; the additional time required to collaborate appropriately with people with lived experience to design, prepare and deliver content [25]; and a lack of university policies and systems to facilitate participation [22, 24]. It is also important to consider that whilst involvement in education may contribute positively to the wellbeing and sense of purpose of people with lived experience, without appropriate preparation, training, support, debriefing, and remuneration this involvement may have negative impacts [21].

The use of storytelling is an engaging pedagogical approach and one technique that enables people with lived experience to participate in the education of health professionals. Storytelling has become an important educative tool in the education of health professionals as it has the power to convey the vulnerability of human experience [8, 21]. The most common use of storytelling involves the person describing their first-person experience of receiving a diagnosis, living with the symptoms of mental ill-health, and their engagement with the mental health system [11, 24]. Effective use of storytelling in learning activities by people with lived experience can enhance learners’ understanding of the experiences of people who have experienced psychological distress or mental ill-health. This approach to education can assist learners to develop skills in empathy and to view people with lived experience first and foremost as people [20]. It also involves consideration of language and descriptors and may challenge the “them and us” concept. Whilst storytelling is considered a powerful learning tool, it has also been suggested it is “a source of vulnerability and potential tokenism” [20, p. 552] if it is not integrated in a careful and collaborative manner.

Effective integration of the knowledge, skills and experiences of people with lived experience may be best achieved through a co-designed approach. The use of a co-design framework advocates for each person to have an “ongoing equal stake, voice and power in the discourse from start to finish, in a bottom-up process” [26, p. 59] and draws on the expertise and experience of clinicians, academics, people with lived experience and other key people [27]. Observing the principles of co-design and acknowledging the benefits and challenges of the process, helps to ensure that each stakeholder contributes to setting the agenda for the process and achieving outcomes in a collaborative way. Roper et al. [19] proposed that collaborative partnerships to co-design and co-produce outcomes requires the ability to hear and respect other perspectives, an awareness of power disparity, the ability to challenge the status quo, and a long-term commitment to advocacy. Tindall et al. [27] identified that co-design to facilitate mental health reform can feel overwhelming but that it is important, rewarding and potentially transformational. In considering the use of storytelling as an approach to education, a co-designed approach ensures the people with lived experience have the agency to tell their story in their own way whilst facilitating achievement of the agreed learning outcomes.

Throughout 2019 and 2020 the Listening to Voices [28] project and Charles Sturt University academic staff worked together to co-design and co-produce a sustainable, education resource. The development of the resource aimed to address feedback from students about a perceived lack of preparation for mental health placements and practice; feedback from people with lived experience about their willingness to make contributions to student education; staff commitment to engage people with lived experience more meaningfully and sustainably in the co-design and delivery of education experiences; and release of new professional competency and accreditation standards that included an increased focus on authentic consumer engagement in courses. The outcome of the collaboration was a free to access, online learning resource that respectfully and artistically presents the lived experiences of four people who have been consumers of the Australian mental health system (https://listeningtovoices.org.au/). The four people who feature in the resource were essential contributors to its design and development. Their shared goals were to make a meaningful contribution to the education of others, to improve understanding of the impact of trauma, to contribute to improving health services, care and support provided to people with similar experiences, and to contribute to the creation of a “community environment in which stigma and discrimination are reduced” [29, p. 7].

The co-designed, free, online education resource includes four short films. Each film shares the journey of one of the experts by experience and all four films address the topics of trauma; the system; stigma and language; hope and transformation. A complementary handbook, collaboratively compiled by the team, is also provided for educators and includes links to additional resources to enhance learning activities and to facilitate deeper learning about topics. The resource was deliberately designed to be flexible; educators are encouraged to consider ways to situate it in learning activities to achieve specific learning outcomes while maintaining respect for the stories that have been shared. For each of the four topics within the stories, broad learning outcomes have been developed by the team (see Table 1).

**Table 1:** Listening to Voices education resource focus topics and associated learning objectives

Topic	Learning objective
Trauma	Learners develop an understanding of the relationship of trauma, including childhood trauma, to health outcomes
The system	Learners reflect on the current mental health service system from those who have a lived experience of it and consider the role of the professional
Stigma and language	Learners will gain an insight into the impact of stigma and relevance of language and will critically reflect on the use and influence of language
Hope and transformation	Learners will develop an attitude of hopefulness and be open to exploring diverse approaches to healing emotional distress

As previously noted, educators play a key role in the development and delivery of educational content and thus obtaining their perspectives about this new learning resource was deemed to be important and relevant to its ongoing development. The purpose of this research project, therefore, was to gain insights into educators’ use of the Listening to Voices online learning resource. More specifically the study aimed to understand the perspectives of educators regarding the resource to:

1. Determine the relevance of the material to meet learning outcomes.
2. Determine the practicality of using the resource;
3. Identify the enablers or barriers to using the resource; and.
4. Identify improvements to enhance the Listening to Voices online learning resource.

## Methods

This research study was reviewed and approved by the Charles Sturt University Human Research Ethics Committee (reference number: H21435). All participants provided informed consent for their survey and/or interview responses to be recorded, collected, analysed, and disseminated.

### Study design

A cross sectional study design was used to survey registered users of the online Listening to Voices education resource. Following the online launch of the resource, advertised widely via various platforms to health, human service and education sectors in Australia, people were invited to register to access and use the resource. At the time of registering to access the education resource, people consented to be contacted for research purposes. At completion of the online survey, participants were asked if they wished to participate in an in-depth interview to provide further feedback about the resource.

It has been recognised that lived experience researchers make a unique contribution to the process and outcomes of research and provide an important link to the broader community [30]. To respect this unique contribution and to enhance the outcomes of this research, the research team included two lived experience researchers who were involved in all stages of the research project. Lived experience researchers were involved in survey question design and wording, interpretation of survey responses and were provided with mentoring to support them to conduct the online, in-depth interviews and to participate in qualitative data analysis.

### Participants and recruitment

People who had registered to use the resource and self-identified their role as “educator” were invited to participate via an email containing the survey link. The email invitation to complete the survey was sent by a member of the research team who was not involved in the development or promotion of the resource thereby minimising the risk of coercion.

Survey participants were asked to provide their email address if they agreed to participate in a follow-on interview. Participants who agreed to participate in a follow-on interview were contacted by a member of the research team to arrange a mutually convenient interview time.

### Data collection

An online survey tool was developed by the research team to address the research questions. Lived experience co-researchers were instrumental in survey development and the choice of terminology used. The survey included 16 questions including demographic questions, closed questions, and open-response questions (see Additional file 1 for the full survey). The online survey was hosted on the Survey Monkey © platform and remained open for three months; participants were provided with one email reminder to complete the survey. The survey was administered by the Spatial Analysis Network (SPAN) at Charles Sturt University. Identifying data were removed by SPAN staff prior to data being made available to the research team for analysis.

Interviews were facilitated by one or both lived experience co-researchers with support from an experienced researcher. An interview guide was developed based on the research questions and informed by the initial analysis of survey data. The aim of the interviews was to explore the survey responses in more depth. All interviews were conducted online using the Zoom platform. Interview length ranged from 22 to 58 min with the average length of interview approximately 42 min. Any potentially identifying information was removed by the research team during the process of transcription and analysis.

### Data analysis

Analysis of survey responses was undertaken to identify patterns of current resource use, implementation in different learning contexts and to identify possible changes to the resource to improve utility. The analysis was descriptive in nature identifying response rates and frequencies as appropriate for each variable allowing trends in the data to be identified. The open reflective questions were analysed qualitatively to identify themes.

The research team used a collaborative, inductive approach to thematic analysis to code and generate themes from the interview data. This process of thematic analysis used the six stages as outlined by Braun and Clarke [31]. The process included: (1) Becoming familiar with the data; (2) Generation of initial codes; (3) Identifying themes; (4) Reviewing the potential themes; (5) Defining the identified themes; and (6) Writing up the findings. Interview data were initially coded independently by all members of the team and then grouped according to themes through a collaborative process involving all members of the research team. The iterative process used to review and code the data aimed to ensure auditability and credibility, and to enhance the trustworthiness of the thematic analysis phase. The final themes from interview data were compared with themes derived from the open-response survey questions and where appropriate, themes were combined.

### Findings

The online survey was sent to 112 registered users who identified as an educator or professional development coordinator. Fifteen invitations to complete the survey received an out of office on extended leave, notice of resignation from position or error reply; 97 surveys successfully reached a registered user. A total of 17/97 completed surveys were returned (approximately 17.5% response rate). Five survey participants agreed to be contacted for an interview.

The findings are presented in two parts. Part one is further divided into two sections; these two sections summarise the findings from the closed ended survey questions relating to the contexts in which the resource was being used, the relevance of the resource to these contexts and the practicality of using the resource for education.

Part two of the findings presents the analysis of the open-ended survey responses and interview data. Four overarching themes were identified from the analysis of this qualitative data, these themes were: (1) scope for application; (2) future resource enhancement; (3) creating safe learning environments; and (4) impact of the resource. The first two themes aligned with and supported the findings from the quantitative data. Two additional themes – creating safe learning environments and the impact of the resource – were identified from the qualitative data. See Table 2 for an overview of the findings.

**Table 2:** Overview of the findings and themes identified

**Findings Part One**
• Learning context and application • Relevance and practicality
**Findings Part Two**
• Scope of application • Future resource enhancement • Creating safe learning environments • Impact of the resource

### Findings part one – quantitative survey data

#### Learning context and application

Survey respondents were employed in a range of health and education settings. Approximately half (n = 8; 47%) were employed in tertiary education settings; one-quarter in the health service sector (n = 4; 24%); with fewer respondents employed in the disability/social service sectors (n = 2; 12%); and a group who identified “other” work contexts (n = 3; 17%).

Most survey respondents (n = 14; 88%) had viewed the resource at the time of completing the survey and half of these had commenced using it in education activities (n = 7); the remainder indicated they were planning to use it. In the tertiary education setting it was being used with undergraduate and masters level students (including public health, health science, nursing, and occupational therapy students). Content had been embedded in courses from first through to third year level, as well as in post-graduate levels of education. In the health and disability/social service sectors the resource had been used with nursing, allied health, medical staff, and medical students on placement. The resource had been used with cohorts as small as two learners to as large as 300. Most educators using the resource were also using the Facilitators’ Guide to support the development and delivery of education activities (n = 6/7; 83%).

#### Relevance and practicality

Survey respondents positively endorsed the relevance of the resource content for meeting the learning needs and outcomes of learners (‘strongly agree’ n = 6; 33%; ‘agree’ n = 8; 50%; ‘partially agree’ n = 3; 17%). All respondents ‘agreed’ or ‘strongly agreed’ that the resource enabled learners to have an increased understanding of the experiences of people living with mental health issues, and similarly all respondents ‘agreed’ or ‘strongly agreed’ that the resource met a gap in education resources regarding people living with mental ill-health.

One of the study objectives was to identify barriers and facilitators to using the resource. Participants who had used the resource reported that having free access to the resource contributed to its utility. The online nature of the resource also enhanced access to and use of the materials. The flexible nature of the resource and the different stories and themes were also perceived to be a strength of the resource enabling educators to choose the story/theme that best aligned with the learners/students and the identified learning outcomes. Respondents noted that the collaborative co-design approach to development of the resource ensured it was both authentic to lived experience, evidence based, academically supported, and professionally presented.

Responses from both the surveys and the interviews demonstrated the resource was being used in a variety of different ways including as part of class activities, as preparation for class activities, as a component of staff induction programs, and to enhance staff professional development activities. It was also being used to achieve a range of specific learning outcomes identified by educators including:

- Developing a better understanding of mental health and mental ill-health;
- Learning about voice hearing and human distress;
- Understanding trauma and the impact of trauma;
- Developing clinical skills for health professionals working in mental health;
- Orientation and induction to particular work settings; and.
- Developing skills for working and communicating with individuals.

Respondents who were using the resource generally reported feeling confident embedding the resource in their teaching (‘strongly agree’ 50%; ‘agree’ 33%; ‘partially agree’ 17%). It was noted that careful planning regarding how the resource would be used was needed. There was a focus on the importance of the educator being familiar with materials prior to using them with learners, and to consider how they would create a safe space for learners.

The responses indicated that all four stories were being used with one story being used more frequently than the other three. The decision to use a particular lived experience story related to alignment with specific learning outcomes, selection of story based on gender, a perception of some stories being more or less confronting for learners to engage with, ease of integrating the story and content into course materials, resonance with learners, and preparation for future work settings.

### Findings part two – qualitative survey and interview data

#### Scope of application

Interview participants discussed how the resource could be adapted to suit specific learning outcomes, a range of learning situations and settings, and could be delivered using a variety of approaches (for example face-to-face or online). Interviewee 3 talked about how they “match the content to the audience…I can do a quick session with discussion…or add the films together for a longer session” highlighting the flexible nature of the resource. In wanting to expose learners to alternatives to the medical model, Interviewee 5 stated how they wanted to “tip the scales towards lived experience, away from the medical model, and this resource allows us to do this.”

Similar to the findings from the survey, interview participants were using the resource with a range of health disciplines. Some participants provided suggestions for other disciplines who they believed would also benefit from engaging with the resource as part of their education:

“I volunteer for the [ambulance service] and hope to get the training as part of their mental health education in the next twelve months…I think the fire brigade is a less obvious target but I may well target them later… the people who developed the Life Education Vans… I will also meet with them to see if they are able to use the product” (Interviewee 3).

### Future resource enhancement

The primary area for future resource enhancement was inclusion of a greater diversity of lived experiences, including contributions from people from culturally diverse backgrounds. In particular, the voice of Indigenous Australian peoples was identified as a gap in currently available education resources. As with survey responses, there were suggestions from the interview participants regarding increasing the diversity of the stories to better reflect contemporary society, for example inclusion of “the experience of someone who is LBGTIQ + and a voice hearer… or an older male person” (Interviewee 3).The resource lists included in the Facilitators’ Guide are predominantly specific to Australia and may be less relevant to international settings; it was suggested that consideration be given to expanding the resource lists.

A range of suggestions were provided to enhance ongoing development of the resource. One interviewee suggested it would be advantageous for the people with lived experience who feature in the resource to be more available for live classroom performance:

“I am reluctant to share someone’s story to that depth without bringing them into the room to elaborate … because that content produces so many questions. It’s too risky for me as an educator to take that position of knowledge and try to answer on behalf of the person” (Interviewee 1).

However, interviewees recognised the time commitment and potential travel this may require, and that this approach would require funding that may be difficult to secure.

While some participants liked the flexibility of the resource and the scope, they had to design how they would use the content within their education sessions and this prompted other participants to suggest that a more structured or modularised approach to the resource would be beneficial. Further development of the Facilitators’ Guide was endorsed along with the inclusion of more references, structured learning activities, and key learning outcomes associated with each story and/or theme.

There was some discussion about the accessibility of the resource – at the time of the interviews users were required to register and then be approved for use. There were mixed feelings about making the resource open access “I would love it to be like an open tool with everyone watching. I don’t think my students need my opinion to engage with it. I don’t think we need an institution to mediate” (Interviewee 1). In response to similar feedback from a range of sources, the resource now has open access to any person who registers.

### Creating a safe learning environment

A recurrent theme arising from the qualitative data related to the need for focused attention on creating a safe learning environment when using the resource. Participants discussed that exploring traumatic human experiences such as those presented in the resource may trigger previous experiences and emotions for some learners and educators: “I acknowledge that the experience in the videos might be similar to something our students and ourselves have experienced” (Interviewee 1). For other learners and educators, it was noted this may be their first exposure to material similar to that covered in the resource. Creating safe environments to explore the range of responses that may occur was highlighted by participants as being an important consideration. The participants acknowledged that some of the content in the resource is confronting, however most participants perceived that exposing learners to this content in a safe environment and providing opportunity for discussion and exploration prior to undertaking a supervised placement or securing employment was better than “letting them loose without this education” (Interviewee 3). The interview participants outlined various strategies they use to facilitate safety, self-care and the care of the learners when using the resource, for example:

“I trust them enough that if it’s uncomfortable and it moves them, they can leave, and they will come back and we will have a dialogue” (Interviewee 1).

“People are asked if they leave the room to give a thumbs up if ok and thumbs down if distressed. I talk to the importance of ‘hearing and listening’ with the heart and how to manage emotions that may happen in response” (Interviewee 3).

“You need to provide time for [learners] to sit with emotion, connect with these emotions and to share and discuss an embodied experience… Run grounding activities after watching the films…look after yourself” (Interviewee 5).

### Impact of the resource

The overwhelming feedback from educators about the resource related to the powerful impact it has on those who engage with it – including both learners and educators:

“There was a lot of emotion in the room. It really rings true…it resonated…it was relatable…it is such a powerful thing. People are gripped – and wow!” (Interviewee 5).

“This really punches the whole point home” (Interviewee 2).

The stories of those with lived experience, coupled with the artistic elements were perceived by educators to make the learning activities impactful and memorable, and the message being presented “hearable.” It was perceived that through using the resource learners improved their understanding of trauma from the perspectives of people with lived experience, were presented with alternatives to deficit discourses, and were encouraged to consider and advocate for diverse approaches to mental health issues. The impact of one of the stories on a student nurse was described:

“Ben’s bullying story was very powerful with one student nurse… After viewing, he was teary, could relate to parts of the story…big attitude adjustment.” (Interviewee 3).

The topics addressed in the resource were perceived to provide opportunities to develop students’ empathy and facilitate students’ use of person-first language and approaches to engagement, as Interviewee 4 stated: “The program works…it builds empathy.” Of importance, the participants perceived that the hope and opportunity for recovery described by people with lived experience challenged commonly held misconceptions and stigma associated with people experiencing mental ill-health.

## Discussion

This evaluation aimed to gain insights into educators’ perceptions of the Listening to Voices online learning resource and to understand their views regarding the relevance, impact and practicality of the resource. Additionally, the evaluation sought to identify aspects of the resource that could be further enhanced. Analysis of the data collected through both surveys and interviews demonstrated the resource is an impactful learning tool that is easily accessible and has broad application. The findings also suggest there is scope for further development of the resource.

The Listening to Voices online films and Facilitators’ Guide provides a suite of co-designed resources to facilitate education about psychological distress, mental ill-health, the impact of trauma, person-centred care, and navigating the mental health system from the perspectives of people with lived experience. Other researchers have supported the value of authentically including people with lived experience in education to reduce stigma, challenge negative attitudes and demystify mental illness [32]. It has also been emphasised that contact with people with lived experience of mental health issues provides opportunities for learners to develop empathy and new perspectives [33, 34]. Difficulties implementing trauma informed care and practice due to a lack of education and training have been identified through research [35, 36]. In a scoping review conducted by McNaughton et al. [37] it was noted that although education about trauma informed care is occurring with health professionals (predominantly nurses), they found minimal evidence to show that people with lived experience were included in this education. The Listening to Voices resource has much to contribute to the reduction of stigma often associated with people experiencing psychological distress and mental ill-health and to increasing the effective implementation of trauma informed care principles. Engagement with the Listening to Voices content has the potential to impact the attitudes and approach of current and future health professionals ensuring they have capacity to participate in critical conversations, and to advocate for consumer leadership and systemic change to mental health services [15]. By raising the awareness of mental health and mental ill-health and challenging the associated stigma and discrimination, as recommended by the World Health Organization [38], the work of the Listening to Voices project aims to advocate for the human rights of people with mental health conditions.

The pedagogical approach used in the Listening to Voices online education resource draws on storytelling methodology using theatre and interview. A co-design framework guided development of the resource, providing the opportunity to privilege people with lived experience and to address underlying power differentials. The stories are presented as unique and legitimate knowledge; in education settings, the stories are juxtaposed with research and practice evidence to create impactful and memorable learning experiences, and to facilitate critical reflection and dialogue. The use of storytelling methodology was perceived by participants in this evaluation to powerfully engage learners and orient them towards an approach of learning *with* rather than *about* people with mental health issues. Participants used the honest and compelling stories included in the Listening to Voices films to augment learning activities resulting in significant changes to learners’ perceptions and attitudes. Previous research has identified the potential for tension when using lived experience as an education strategy [10]. Arblaster et al. [12] found that although the priorities of people with lived experience and educators may be different, both have a place in education and the aim should be to bring both perspectives together to inform curriculum. The participant responses regarding use of the Listening to Voices resource demonstrated how different forms of evidence and knowledge can be successfully embedded into curriculum to enhance learning and create change whilst simultaneously retaining respect for human experience.

The responses of some participants suggested that not all were comfortable with embedding the content contained in the Listening to Voices resource into learning activities. Other participants in this evaluation highlighted the importance of ensuring educator familiarity with the content and thoughtfully creating safe learning spaces when using the resource. It was noted that both people with lived experience and educators require support to effectively use this collaborative approach to education in a manner that is respectful and sustainable. Dorozenko & colleagues [18, p. 916] have previously found it is important to build the capacity of people with lived experience to share their story in an educative way as well as strengthening “the ability of academics to work with lived experience in broad and value-based ways.” Despite the potential for discomfort and the perceived barriers to using a co-designed approach to the inclusion of people with lived experience in education, “it is essential that students see and hear from people who can share their stories of recovery from mental ill-health and come to understand first-hand that people can and do recover” [18, p. 906]. This approach to education also provides important opportunities to challenge the stigma, fear and negative attitudes associated with mental illness [18, 20, 23].

The straightforward and free online access to the Listening to Voices resource was a factor facilitating its use. Most participants were aware of the benefits, and in some cases the requirement of professional bodies, to engage with people with lived experience to educate staff and/or students. However, for some participants institutional barriers including a lack of funding prohibited them doing this in a meaningful, respectful, and sustainable manner. It has been recommended that “mental health awareness needs to be integrated into all elements of health and social policy, health-system planning, and health-care delivery” [39, p. 870], and education [18]. The Listening to Voices resource provides educators and learners with access to co-designed content that prioritises the voices of people with lived experience and that contributes to improved awareness of mental health issues. It is, however, worth noting that participation of people with lived experience in education should extend beyond one-off arrangements where individuals tell their stories to include partnership in design, planning, delivery and evaluation of education programs [6, 8, 11]. The Listening to Voices resource is therefore one strategy in what should be a comprehensive approach to facilitating the engagement of people with lived experience. Other strategies to promote success in this space include commitment to authentic partnerships, support, and autonomy [3, 40], formal processes and clear guidelines to ensure people with lived experience are authentically involved in decisions and ongoing design and delivery [23], and allocation of finances and time to ensure the sustainability of arrangements [25].

### Strengths and limitations

One of the strengths of this research is the involvement of people with lived experience as members of the research team who participated in the design and implementation of the resource evaluation. Including lived experience researchers ensured their unique contributions were respected and also enhanced the outcomes of this research through the inclusion of different perspectives. Another strength of the research design was that while all registered users were invited to participate in the evaluation, the survey was deliberately designed using skip logic strategies to ensure that only people who had used the resource in educational activities responded to questions about application; this approach provided rich, experiential data.

The evaluation has several limitations. The low number of participants in both the survey and interviews has resulted in a limited range of perspectives regarding the use of the resource as an educative tool being captured. All participants had watched and had either used or had active plans to use the resource and thus it could be assumed they perceived the resource favourably. It would be beneficial to speak with those people who have registered to use the resource and who have decided not to use it, to gain their perspectives. The perspectives captured in this evaluation are those of educators; developing a fuller understanding of the impact of this resource as an impactful education tool by including the perspectives of learners and the people who tell their stories is recommended. The authors strongly advocate for the inclusion of lived experience researchers and believe their perspectives enhanced this evaluation, however it is possible the facilitation of the interviews by one of the lived experience experts from the online resource may have created hesitancy in the responses of some participants or a tendency to provide overly positive feedback.

## Conclusion

The educators who participated in this evaluation highlighted the relevance, impact, and practicality of the Listening to Voices online education resource. The flexibility of and free access to the resource were perceived to improve utility. The findings of this evaluation have demonstrated that involving people with lived experience in the education of healthcare students and professionals is perceived by educators to assist in developing essential skills for collaborative, compassionate, and person-centred care. Implementation of co-design principles and the use of creative pedagogical approaches, such as storytelling and theatre, can contribute to the development of resources to support education experiences that foreground lived experience. This approach was perceived by educators to be an impactful way to educate health professional students and staff about mental health issues and human distress. There is scope for further development of and research into this resource.

### Electronic supplementary material

Below is the link to the electronic supplementary material.

**Supplementary Material 1:** Listening to Voices online learning resource: Educator Survey

## Data Availability

Data and materials related to this study can be requested by contacting Dr Tracey Parnell (tparnell@csu.edu.au). All of the material is owned by the authors and/or no permissions are required.

## References

[1] Sunkel C, Sartor C (2022). Perspectives: involving persons with lived experience of mental health conditions in service delivery, development and leadership. Br J Psychiatry Bull.

[2] Arblaster K, Mackenzie L, Buus N, Chen T, Gill K, Gomez L, Hamilton D, Hancock N, McCloughen A, Nicholson M, Quinn Y, River J, Scanlan J, Schneider C, Schweizer R, Wells K. Co-design and evaluation of a multidisciplinary teaching resource on mental health recovery involving people with lived experience. Aust Occup Ther J. 2023;1–12. 10.1111/1440-1630.12859. 10.1111/1440-1630.12859 36704991

[3] Happell B, Roper C (2003). The role of a mental health consumer in the education of postgraduate psychiatric nursing students: the students’ evaluation. J Psychiatr Ment Health Nurs.

[4] Knaak S, Mantler E, Szeto A (2017). Mental illness-related stigma in healthcare. Healthc Manage Forum.

[5] Logan A, Yule E, Hughes J, Peters D, Hadley M, Betts B, Jones L, Froude E (2022). The impact of face-to-face mental health consumer-led teaching on occupational therapy student empathy levels: two group comparison design. Aust Occup Ther J.

[6] Occupational Therapy Council (Australian and New Zealand). (2018). *Accreditation standards for entry level occupational therapy education programs*. Occupational Therapy Council. http://www.occupationaltherapyboard.gov.au/documents/default.aspx?record=WD13%2F11077&dbid=AP&chksum=4Z4x90ijo8T7bnTDGxKBPQ%3D%3D.

[7] Hardy S, Malby R, Hallett N, Farooq A, Chamley C, Young G, White X, Turner W (2018). Introducing a people’s academy into higher education. High Educ Skills Work-Based Learn.

[8] Horgan A, Donovan MO, Doody R, Savage E, Dorrity C, O’Sullivan H, Goodwin J, Greaney S, Biering P, Bjornsson E, Bocking J, MacGabhann L, Russell S, Griffin M, van der Vaart KJ, Allon J, Granerud A, Hals E, Pulli J, Vatula A, Ellilä H, Lahti M, Happell B (2021). Improving service user involvement in mental health nursing education: suggestions from those with lived experience. Issues Ment Health Nurs.

[9] Beresford P, Croft S (2001). Service users’ knowledges and the social construction of social work. J Social Work.

[10] Happell B, Gordon S, Sharrock J, O’Donovan A, Warner T (2022). What’s she doing here?’ Overcoming barriers to the implementation of Expert by Experience positions in academia. Aust Occup Ther J.

[11] Arblaster K, Mackenzie L, Willis K (2015). Mental health consumer participation in education: a structured literature review. Aust Occup Ther J.

[12] Arblaster K, Mackenzie L, Willis K (2018). Learning from consumers: an eDelphi study of Australian mental health consumers’ priorities for recovery-oriented curricula. Aust Occup Ther J.

[13] Happell B, Wynaden D, Tohotoa J, Platania-Phung C, Byrne L, Martin G, Harris S. (2015). Mental health lived experience academics in tertiary education: the views of nurse academics. Nurse Education Today,35(1), 113–7. 10.1016/j.nedt.2014.07.006. 10.1016/j.nedt.2014.07.006 25112925

[14] Classen B, Tudor K, Johnson F, McKenna B (2021). Embedding lived experience expertise across the mental health tertiary education sector: an integrative review in the context of Aotearoa New Zealand. J Psychiatr Ment Health Nurs.

[15] Happell B, O’Donovan A, Sharrock J, Warner T, Gordon S (2021). They are a different breed aren’t they? Exploring how experts by experience influence students through mental health education. Int J Ment Health Nurs.

[16] Ridley S, Martin R, Mahboub L (2017). Learning from mental health lived experience and the influence on students’ practice. Australian Social Work.

[17] Towle A, Godolphin W (2011). A meeting of experts: the emerging roles of non-professionals in the education of health professionals. Teach High Educ.

[18] Dorozenko K, Ridley S, Martin R, Mahboub L (2016). A journey of embedding mental health lived experience in social work education. Social Work Education.

[19] Roper C, Hopkins F, Houghton J. Who’s got the wheel? – consumer leadership and co-production in the training of mental health clinicians. Health Issues Summer. 2014;11334–7. 10.3316/informit.834953258220146.

[20] Happell B, Bennetts W, Harris S, Platania-Phung C, Tohotoa J, Byrne L, Wynaden D (2015). Lived experience in teaching mental health nursing: issues of fear and power. Int J Ment Health Nurs.

[21] Happell B, Bennetts W (2016). Triumph and adversity: exploring the complexities of consumer storytelling in mental health nursing education. Int J Ment Health Nurs.

[22] Basset T, Campbell P, Anderson J (2006). Service user/survivor involvement in mental health training and education: overcoming the barriers. Social Work Education.

[23] Happell B, Bennetts W, Platania-Phung C, Tohotoa J (2015). Consumer involvement in mental health education for health professionals: feasibility and support for the role. J Clin Nurs.

[24] Happell B, Platania-Phung C, Byrne L, Wynaden D, Martin G, Harris S (2015). Consumer participation in nurse education: a national survey of Australian universities. Int J Ment Health Nurs.

[25] Scanlan JN, Logan A, Arblaster K, Haracz K, Fossey E, Milbourn BT, Pepin G, Machingura T, Webster JS (2020). Mental health consumer involvement in occupational therapy education in Australia and Aotearoa New Zealand. Aust Occup Ther J.

[26] Rosen A, Holmes DJ (2023). Co-leadership to co-design in mental health-care ecosystems: what does it mean to us?. Leadersh Health Serv.

[27] Tindall RM, Ferris M, Townsend M, Boschert G, Moylan S (2021). A first-hand experience of co‐design in mental health service design: opportunities, challenges, and lessons. Int J Ment Health Nurs.

[28] Listening to Voices. (2023). *Listening to Voices* https://listeningtovoices.org.au/.

[29] National Mental Health Commission. (2022). *Vision 2030 for mental health and suicide prevention in Australia* https://www.mentalhealthcommission.gov.au/projects/vision-2030.

[30] Banfield M, Randall R, O’Brien M, Hope S, Gulliver A, Forbes O, Morse AR, Griffiths K (2018). Lived experience researchers partnering with consumers and carers to improve mental health research: reflections from an Australian initiative. Int J Ment Health Nurs.

[31] Braun V, Clarke V. Successful qualitative research: a practical guide for beginners. Sage; 2013.

[32] Vigo D (2016). The health crisis of mental health stigma. Lancet.

[33] Byrne L, Happell B, Welch T, Moxham LJ (2013). Things you can’t learn from books: teaching recovery from lived experience. Int J Ment Health Nurs.

[34] O’Reilly CL, Bell JS, Chen TF (2011). Mental health consumers and caregivers as instructors for health professional students: a qualitative study. Soc Psychiatry Psychiatr Epidemiol.

[35] Isobel S, Wilson A, Gill K, Schelling K, Howe D (2020). What is needed for a trauma-informed mental health service in Australia? Perspectives of clinicians and managers. Int J Ment Health Nurs.

[36] New South Wales Agency for Clinical Innovation. (2019). *Evidence series: trauma-informed care and mental health in NSW* https://aci.health.nsw.gov.au/__data/assets/pdf_file/0008/561977/ACI-Trauma-informed-care-and-mental-health-in-NSW-evidence-series.pdf.

[37] McNaughton KM, Isobel S, Phelan L, Quilty E (2022). Trauma-informed training and education for professionals in Australia: a scoping review. J Mental Health Train Educ Pract.

[38] World Health Organization. (2019). *Mental Health* https://www.who.int/health-topics/mental-health#tab=tab_1.

[39] Prince M, Patel V, Saxena S, Maj M, Maselko J, Phillips MR, Rahman A (2007). No health without mental health. Lancet.

[40] Happell B, Roper C (2009). Promoting genuine consumer participation in mental health education: a consumer academic role. Nurse Educ Today.

