# Supplementary material for: Lived experience narratives in health professional education: educators’ perspectives of a co-designed, online mental health education resource
Source: BMC Med Educ. 2023 Dec 12;23:946. doi: 10.1186/s12909-023-04956-0 (PMC10717857; doi:10.1186/s12909-023-04956-0)
Supplement: Supplementary file 1 — Supplementary Material 1: Listening to Voices online learning resource: Educator Survey [file 12909_2023_4956_MOESM1_ESM.pdf]

Charles Sturt University

Listening to Voices Online Learning Resource

Invitation and Participant Information

Research invitation

You are invited to participate in a research study being conducted by a team of researchers from Charles Sturt University, Three Rivers Department of Rural Health, Gateway Health, and Intervoice.

Project Title

Listening to Voices Online Education Resource – Educators' and professional development coordinators' feedback

Research team

Lead Researcher:
Dr Tracey Parnell - Associate Head of School & Head of Discipline – Occupational Therapy, School of Allied Health, Exercise & Sports Sciences, Charles Sturt University
Co-Researchers:
Ms Kate Fiske - CHIPS Coordinator & Listening to Voices Project Manager, Gateway Health
Ms Sarah Sewell - Project Team Member - Listening to Voices
Ms Kellie Stastny - Chair - Intervoice
Ass Prof Melissa Nott - Principal Research Fellow, Three Rivers Department of Rural Health, Charles Sturt University

Before you decide whether you wish to participate in this study, it is important for you to understand why the research is being done and what it will involve. Please take the time to read the following information carefully.

What is the purpose of this study?
The purpose of this study is to gather the perspectives of educators and professional development coordinators on the Listening to Voices Online learning resource to:

- Determine the relevance of the material for meeting learning outcomes;
- Determine the practicality of using the resource;
- Identify the enablers or barriers to using the resource; and
- Identify improvements to enhance the L2V online learning resource

The findings from this study will inform subsequent versions of the online learning resource and supporting materials.

Why have I been invited?
You have been invited to take part in the study because you have registered as an educator or professional development coordinator to use the Listening to Voices online learning resource.

What does this study involve?
If you agree to participate, you are asked to complete this survey about your use or planned use of the Listening to Voices online learning resource. The survey includes questions about which parts of the resource you have used or plan to use and how you have used or plan to use these parts; there are also opportunities for you to suggest ways to improve and develop this resource.

The survey should take no more than 15 minutes to complete. There are no right or wrong answers to the questions and your honesty in reporting your experience and suggestions is appreciated.

What will happen to the information that I give you?
The information you provide will be used by the Listening to Voices project team to continue to develop and improve the resource; your suggestions may also be used to develop new resources. The results of this research will also be published in journals and presented at conferences and may be shared through social media outlets. Your survey responses will be anonymous and therefore you will not be able to be identified in any publications or presentations.

How will my confidentiality be protected?
It will not be possible to identify you from your survey. Any potentially identifying information from surveys, such as IP addresses, will be deleted by the survey administrator prior to the research team receiving the survey responses. The survey responses will be retained securely for 5 years in line with Charles Sturt University policies and procedures.

Are there any benefits or risks to me in taking part in this study?
Providing your feedback on this resource can help to support further development of the resource and the importance of integrating lived experience in learning experiences. The opportunity for reflection may also provide you with time and space to deepen your learning and engagement with the resource.

You may find content in the resource to be personal and sensitive in nature. The survey questions may also prompt you to reflect on your teaching and learning strategies and the effectiveness of these. All the responses you provide will be anonymous and if you feel uneasy with any of the questions, you can leave them out or stop completing the survey.

The information you provide will be treated in a respectful and confidential manner. The survey data will be anonymous, no-one (not even the researchers) will be able to associate you with your survey responses.

If you become distressed while completing the survey or interview and require support, you may wish to access one of the following services:

- Lifeline: 131114
- Beyond Blue: 1300 224 636
- Blue Knot: 1300 657 380

How is the study being paid for?
This research is not being funded. Staff members of the School of Allied Health, Exercise and Sports Sciences at Charles Sturt University, Gateway Health, Three Rivers Department of Rural Health at Charles Sturt University and Intervoice will complete this study as part of their usual work. The Rural Health Multidisciplinary Training Scheme which is funded by the Federal Government Department of Health, will provide funding to remunerate the lived experience researchers.

What if I don’t want to take part in this study?
Completion of the survey is voluntary; there is no obligation to complete the survey. As a registered user you will still be able to access the Listening to Voices online learning resource even if you decide not to complete the survey. You also do not need to answer every question. If you change your mind once you have submitted the survey you will not be able to withdraw your responses as your survey is anonymous and any identifying data will have been removed.

What should I do if I want to discuss this study further before I decide?
If you would like further information, please contact: Associate Professor Melissa Nott (mnott@csu.edu.au).

Who should I contact if I have concerns about the conduct of this study?
Charles Sturt University’s Human Ethics Committees have approved this project [Protocol number: 21435]. If you have any complaints or reservations about the ethical conduct of this project, you may contact the Committee.

Human Research Ethics Committee
Phone: 02 6933 4213
Email: ethics@csu.edu.au

Any issues you raise will be treated in confidence and investigated fully and you will be informed of the outcome.

Thank you for taking the time to read this information and consider participating in the study.
If you wish to keep a copy of this information for your records, please copy and paste these information pages into a document or print screen.

Charles Sturt University

Listening to Voices Online Learning Resource

Consent

* 1. By completing this questionnaire, you are indicating that you:

- **Have read and understood the information about this research study;**
- **Have had any questions answered to your satisfaction**
- **Understand that if you have any additional questions you can contact the researchers;**
- **Understand that any answers you provide may be used anonymously in the research study**

○ I agree

○ No, I do not agree

Charles Sturt University

Listening to Voices Online Learning Resource

2. Which sector best describes where you currently work?

- ◯ Education
- ◯ Health
- ◯ Disability / Social Services
- ◯ Other (please specify)

3. Have you viewed the Listening to Voices online learning resource?

- ◯ Yes
- ◯ No

Charles Sturt University

Listening to Voices Online Learning Resource

4. What have been the barriers to viewing the resource?

Charles Sturt University

Listening to Voices Online Learning Resource

5. Are you using the Listening to Voices online learning resource in your teaching?

- ◯ Yes
- ◯ No

Charles Sturt University

Listening to Voices Online Learning Resource

6. Do you intend to use the resource in the future?

- ◯ Yes
- ◯ No

Charles Sturt University

Listening to Voices Online Learning Resource

7. Please elaborate on why you do not intend to use the resource in the future

Charles Sturt University

Listening to Voices Online Learning Resource

8. Please tell us some more about the setting/s in which you have used the resource.
Please include as much detail as possible e.g. tertiary/secondary education setting, health organisation, employment setting, etc.

Charles Sturt University

Listening to Voices Online Learning Resource

9. Please tell us some more about the people/students/learners you have used the resource with:

Number of students/learners:

Course/s/Professions:

Year level (if appropriate):

Nature of subject/Professional development session:

Charles Sturt University

Listening to Voices Online Learning Resource

Stories

10. Which story(ies) in the resource have you used, or do you intend to use, in your teaching?
(Please select all that apply)

- ☐ Ben's story
- ☐ Jain's story
- ☐ Kelly's story
- ☐ Sarah's story
- ☐ None of the stories

Charles Sturt University

Listening to Voices Online Learning Resource

Ben's story

11. Why have you used, or why do you intend to use, Ben's story?

Charles Sturt University

Listening to Voices Online Learning Resource

Jain's story

12. Why have you used, or why do you intend to use, Jain's story?

Charles Sturt University

Listening to Voices Online Learning Resource

Kelly's story

13. Why have you used, or why do you intend to use, Kelly's story?

Charles Sturt University

Listening to Voices Online Learning Resource

Sarah's story

14. Why have you used, or why do you intend to use, Sarah's story?

Charles Sturt University

Listening to Voices Online Learning Resource

The remaining questions relate to educators who HAVE used the Listening to Voices resource.

15. Have you used the resource?

- ◯ Yes
- ◯ No

Charles Sturt University

Listening to Voices Online Learning Resource

16. When preparing your teaching, did you use the Facilitators' Guide to assist you?

- ◯ Yes
- ◯ No

Charles Sturt University

Listening to Voices Online Learning Resource

17. What did you find useful or helpful in the Facilitators' Guide?

18. Please provide any suggestions for improving the Facilitators' Guide:

Charles Sturt University

Listening to Voices Online Learning Resource

Please read the following statements and select the number that is most applicable to you

19. The content of the resource met the learning needs of my students/staff.

Strongly disagree 1	2	3`	4	5	6	Strongly agree 7
○	○	○	○	○	○	○

20. The resource helped my students/staff gain an increased understanding of the experiences of people living with mental health issues.

Strongly disagree 1	2	3`	4	5	6	Strongly agree 7
○	○	○	○	○	○	○

21. The resource met a gap in education resources regarding people living with mental health issues.

Strongly disagree 1	2	3`	4	5	6	Strongly agree 7
○	○	○	○	○	○	○

22. I feel confident using the Listening to Voices resource as part of my teaching.

Strongly disagree 1	2	3`	4	5	6	Strongly agree 7
○	○	○	○	○	○	○

23. If you wish to explain any of your answers in more detail please comment in the box below:

Charles Sturt University

Listening to Voices Online Learning Resource

24. Please identify any factors which helped you use the resource:

25. Please identify any barriers you have encountered when using the resource:

Charles Sturt University

Listening to Voices Online Learning Resource

26. We are eager to hear any other feedback you would like to provide about this resource:

Charles Sturt University

Listening to Voices Online Learning Resource

27. We would like to talk with some people in more detail about their experience of using this resource.
Would you be willing to participate in an interview?

- ◯ Yes
- ◯ No

Charles Sturt University

Listening to Voices Online Learning Resource

28. Thank you for indicating you are willing to participate in an interview.

To enable one of the research team to contact you to arrange an interview time, please provide your contact details below.
(Please note that these details will not be linked to your survey responses)

First Name:

Email:

Phone:

Charles Sturt University

Listening to Voices Online Learning Resource

Thank you for your feedback and comments.
Your input will be used to continue to develop the resource.

Please click on the '**Done**' button below to finalise your survey.
